# Association between advanced maternal age and maternal and neonatal morbidity: A cross-sectional study on a Spanish population

**DOI:** 10.1371/journal.pone.0225074

**Published:** 2019-11-26

**Authors:** Ana Casteleiro, María Paz-Zulueta, Paula Parás-Bravo, Laura Ruiz-Azcona, Miguel Santibañez

**Affiliations:** 1 Hospital Universitario de Basurto, Bilbao, Spain; 2 Department of Nursing, University of Cantabria, Cantabria, Spain; 3 IDIVAL, GRIDES, Cantabria, Spain; 4 IDIVAL, Grupo de Investigación en Enfermería, Cantabria, Spain; Ospedale dei Bambini Vittore Buzzi, ITALY

## Abstract

**Background and objective:**

Over recent decades, a progressive increase in the maternal age at childbirth has been observed in developed countries, posing a health risk for both women and infants. The aim of this study was to analyze the association between advanced maternal age (AMA) and maternal and neonatal morbidity.

**Material and methods:**

A cross-sectional study of 3,315 births was conducted in the north of Spain in 2014. We compared childbirth between women aged 35 years or older, with a reference group of women aged between 24 and 27 years. AMA was categorized based on ordinal ranking into 35–38 years, 39–42 years, and >42 years to estimate a dose-response pattern (the older the age, the greater the risk). As an association measure, crude and adjusted Odds Ratios (OR) were estimated by non-conditional logistic regression and 95% Confidence Intervals (95%CI) were calculated.

**Results:**

Repeated abortions were more common among women of AMA in comparison to pregnant women aged 24–27 years (reference group): adjusted OR = 2.68; 95%CI (1.52–4.73). A higher prevalence of gestational diabetes was also observed among women of AMA, reaching statistical significance when restricted to first time mothers: adjusted OR = 8.55; 95%CI (1.12–65.43). In addition, the possibility of an instrumental delivery was multiplied by 1.6 and the possibility of a cesarean by 1.5 among women of AMA, with these results reaching statistical significance, and observing a dose-response pattern. Lastly, there were associations between preeclampsia, preterm birth (<37 weeks) and low birthweight, however without reaching statistical significance.

**Conclusion:**

Our results support the association between AMA and suffering repeated abortions. Likewise, being of AMA was associated with a greater risk of suffering from gestational diabetes, especially among primiparous women, as well as being associated with both instrumental deliveries and cesareans among both primiparous and multiparous women.

## Introduction

The average age at which women give birth has been steadily increasing over recent decades, especially in developed countries. Several factors influence the rising age of motherhood. Thus, the parallel increase in the age of emancipation, youth unemployment and the longer periods of education, together with the penalization that women suffer in their professional career when they have children, and the lack of measures to reconcile family and support maternity are among the main reasons for this phenomenon. In addition, the changes in society, lifestyle and work priorities, the improved accessibility and advancements in the field of assisted reproduction methods, have also helped to normalize motherhood at progressively older ages [1–3].

This increase in maternal age is a worldwide phenomenon. In Canada, there has been an increase in the percentage of births from women aged between 35–39 years, ranging from 4.7% in 1982 to 14.1% in 2002 [4]. In the UK, a similar phenomenon has been observed: in 1992, 12% of women who gave birth were approximately 35 years old, compared to 20% in 2016 [5]. Likewise, in the USA, birth rates among women of advanced maternal age (AMA) have increased 12% from 2007 to 2016 [3]. In 2016, the birth rate of women aged between 35–39 years was 52.7 for every 1000 women; the highest rate reported since 1962 while, for women aged between 40–44 years, a birth rate of 11.4 for every 1000 women was registered; the highest since 1966 [6].

In Spain, the crude birth rate has decreased by 10.6 points from 1976 to 2017, and the average age of women at childbirth has increased from 28.5 years in 1976 to 32.1 in 2017. The average age of first-time mothers has also increased, from 25.25 in 1975 to 30.9 in 2017. In Cantabria (Spain), the mean age of women at childbirth in 2017 was 32.5 [7].

This progressive increase in maternal age poses a considerable health risk for women. Advanced maternal age is defined as childbearing in a woman over 35 years of age [2, 8–10]. Many studies have reported an association between AMA and a greater incidence of gestational diabetes [2, 5,11,12], gestational high blood pressure [5], an increase in cesarean sections [10–14], induced births [11,12], instrumental deliveries [14] and abortions [1].

Regarding neonatal outcomes, AMA is related with an increase of preterm births [5,15], intrauterine growth restriction (IUGR) [5], a greater incidence of intrauterine fetal death [5] and a low birth weight [9,15].

An important aspect worth considering in women with AMA is the difference in maternal and neonatal risk according to the woman’s parity. Thus, primiparous women with AMA are considered to be at highest risk of complications [2,8]. Primiparous women with AMA have a greater risk of pre-eclampsia [16], HELLP syndrome [16], instrumental birth [2,5]; urgent cesarean [2,5], fetal growth retardation [16], low birthweight [2,8] and perinatal death [5,8,16].

The aim of this study was to analyze the association between AMA and maternal and neonatal morbidity at a regional public hospital located in Cantabria, Spain.

## Materials and methods

A cross sectional study was performed. The total number of births that took place at the Marqués de Valdecilla University Hospital (MVUH) from January 1 to December 31, 2014 was registered (n = 3,315 births). Information concerning each birth was gathered based on the register of births at the MVUH. Qlikview computer applications were used to compare or complete the information in cases where the record did not appear in the register.

The variables gathered were: maternal age at the time of birth, gestational diabetes, high blood pressure without preeclampsia, preeclampsia, cesarean sections, parity (primiparous or multiparous), degree of perineal tear, previous abortion, fetal death (prior to delivery), birth weight and gestational age. According to established definitions, AMA was defined as women ≥ 35 years at delivery [2, 8, 9, 10]. The reference group was restricted to women aged between 24 and 27 years old [2]. This age range encompasses women at a lower risk of maternal and neonatal morbidity [2, 4, 10–13, 17–20], excluding very young women (i.e. women under the age of 24 years) which, as in the case of AMA, may also be considered a risk factor for pregnancy and birth [3]. Additionally, AMA was subdivided into women of between 35–38 years, 39–42 years and >42 years. This is similarly based on published research studies which highlight the greater maternal and neonatal risk among women of a more AMA [21,22].

Birth weight was categorized as low birth weight (less than 2500 grams) or fetal macrosomia (more than 4000 grams). In addition, we created a variable for neonatal weight according to gestational week and gender. We classified ‘small for gestational age’ using the <10^th^ percentile of a previously published population-based reference, and we classified ‘large for gestational age’ using the >90^th^ percentile of a previously published population-based reference [23–25].

The gestational age was categorized as late preterm (less than 37 weeks) and premature (less than 34 weeks).

### Statistical analysis

The data analysis incorporated an initial descriptive analysis. For the categorical and discrete variables, we estimated proportions with their corresponding 95% confidence intervals. This was according to the Wilson method, and used the chi-squared Pearson’s test for comparisons. Alternatively, we used the Fisher’s exact test whereby more than 20% of the fields presented a number of expected cases less than, or equal to, five. For the continuous variables, we estimated the mean and the standard deviation or, in the case of asymmetric distributions, the median and interquartile range. The Shapiro-Wilk test was used to determine the normality of the distributions. Comparisons for continuous variables were performed using the Student’s t-test or the Mann-Whitney U test when appropriate. As the association measure, crude and adjusted odds ratios (OR) were estimated by non-conditional logistic regression computing a 95% confidence interval (95%CI). The following predefined confounding variables were included in the models, when appropriate: parity (primiparous vs multiparous), immigrant status (native vs immigrant)[26], history of previous cesarean sections (no vs yes), onset of labor (spontaneous vs induced).

The alpha error was set at 0.05 and all the p values were bilateral. All statistical analyses were performed using the SPSS v22.0 package by IBM and Stata 13.0.

### Ethical considerations

The research protocol for this study was approved by the Clinical Research Ethics Committee in Cantabria. The data were anonymized and treated confidentially according to the personal data protection legislation in place.

## Results

In 10 of the 3315 total births under study, it was not possible to identify the maternal age at the time of birth. Table 1 summarizes the general characteristics of the study population, including information regarding maternal age (n = 3305 births). The mean maternal age of the sample was 32.52 years [SD = 5.14 years]. Of the total number of births 36.8% (n = 1216) were women of AMA (≥ 35 years). The group comprising women aged between 24 and 27 years represented 9.25% (n = 306) of the total sample. Up to 55.5% of deliveries (n = 1835) were in primiparous women. Up to 6.5% of pregnant women (n = 215) had a background of repeated abortions (more than one abortion). In 2.6% of births (n = 86), pregnant women developed gestational diabetes while 1.8% (n = 61) of pregnant women developed preeclampsia. The global prevalence of instrumental deliveries and cesareans was 35.2 and 22.6%, respectively.

**Table 1:** General characteristics of the population under study according to the maternal age at the time of birth.

	<24		24–27		28–34		≥35		Total		
	n = 201	% *	n = 306	% *	n = 1582	% *	n = 1216	% *	n = 3305	% *	p
Nationality											
Autochthonous	133	66.2	225	73.5	1373	86.8	1103	90.7	2834	85.7	
Immigrant	68	33.8	81	26.5	209	13.2	113	9.3	471	14.3	< 0.001
Obstetric history											
Multiparous women (>1)											
no	155	77.1	204	66.7	961	60.7	515	42.4	1835	55.5	
yes	46	22.9	102	33.3	621	39.3	701	57.6	1470	44.5	< 0.001
Repeated abortions											
No abortion	152	75.6	219	71.6	1211	76.5	806	66.3	2388	72.3	
1 abortion	43	21.4	72	23.5	301	19.0	286	23.5	702	21.2	
> 1 abortion	6	3.0	15	4.9	70	4.4	124	10.2	215	6.5	< 0.001
Previous cesarean											
no	197	98.0	286	93.5	1490	94.2	1066	87.7	3039	92.0	
yes	4	2.0	20	6.5	92	5.8	150	12.3	266	8.0	< 0.001
Onset of labor											
Spontaneous	142	70.6	190	62.1	986	62.3	781	64.2	2099	63.5	
Induced	59	29.4	116	37.9	595	37.6	435	35.8	1205	36.5	0.119
Maternal morbidity											
Gestational diabetes											
no	200	99.5	302	98.7	1544	97.6	1173	96.5	3219	97.4	
yes	1	0.5	4	1.3	38	2.4	43	3.5	86	2.6	0.019
HTN composite											
no	197	98.0	296	96.7	1506	95.2	1167	96.0	3166	95.8	
yes	4	2.0	10	3.3	76	4.8	49	4.0	139	4.2	0.202
HBP without preeclampsia											
no	198	98.5	299	97.7	1523	96.3	1186	97.5	3206	97.0	
yes	3	1.5	7	2.3	59	3.7	30	2.5	99	3.0	0.103
Preeclampsia											
no	200	99.5	303	99.0	1550	98.0	1191	97.9	3244	98.2	
yes	1	0.5	3	1.0	32	2.0	25	2.1	61	1.8	0.278
Instrumental birth											
no	144	71.6	199	65.0	1039	65.7	760	62.5	2142	64.8	
Yes	57	28.4	107	35.0	543	34.3	456	37.5	1163	35.2	0.058
Cesarean section											
No	174	86.6	238	77.8	1248	78.9	898	73.8	2558	77.4	
yes	27	13.4	68	22.2	334	21.1	318	26.2	747	22.6	< 0.001
Severe tear (3rd or 4th degree)											
no	173	99.4	238	99.2	1230	99.7	887	99.8	2528	99.6	
yes	1	0.6	2	0.8	4	0.3	2	0.2	9	0.4	0.522
Neonatal morbimortality											
Gestational age <37 weeks											
no	182	91.5	285	93.1	1470	93.0	1103	90.9	3040	92.2	
yes	17	8.5	21	6.9	110	7.0	110	9.1	258	7.8	0.190
Gestational age <34 weeks											
no	194	97.5	298	97.4	1547	97.9	1180	97.3	3219	97.6	
yes	5	2.5	8	2.6	33	2.1	33	2.7	79	2.4	0.739
Apgar <7 at 5 min**											
no	199	99.5	300	99.0	1559	99.0	1179	98.4	3237	98.8	
yes	1	0.5	3	1.0	15	1.0	19	1.6	38	1.2	0.345
Apgar <4 at 5 min**											
no	200	100.0	303	100.0	1570	99.7	1193	99.6	3266	99.7	
yes	0		0		4	0.3	5	0.4	9	0.3	0.513
Birth weight**											
normal birth weight	162	91.5	238	88.5	1298	89.0	992	91.2	2690	89.9	
low birth weight	9	5.1	10	3.7	66	4.5	49	4.5	134	4.5	
fetal macrosomia	6	3.4	21	7.8	94	6.4	47	4.3	168	5.6	0.131
normal for gestational age	152	85.9	226	84.0	1238	85.0	921	84.9	2537	84.9	
small for gestational age	3	1.7	6	2.2	25	1.7	26	2.4	60	2.0	
large for gestational age	22	12.4	37	13.8	193	13.3	138	12.7	390	13.1	0.927
Intrauterine fetal death											
No	201	100.0	306	100.0	1579	99.8	1209	99.4	3295	99.7	
Yes	0		0		3	0.2	7	0.6	10	0.3	0.157

* % valid without missing data

** only live fetuses

HTN Hypertension

HBP High Blood Pressure

A low birth weight and premature births occurring at less than 37 weeks were the only adverse results that were more prevalent in the group of <24 years when compared to the group selected as the reference category (24–27 years).

With the exception of the percentage of induced births, the percentage of severe tears (3rd and 4th degree tears) and birth weight > 4000 kg (fetal macrosomia), the remaining adverse effects studied were more prevalent in the group of AMA when compared to the group selected as the reference category.

Table 2 displays the associations between maternal and neonatal morbidity and AMA. Regarding the women’s medical background, repeated abortions were 2.20 times more frequent in women of AMA; 95%CI (1.27–3.82). When adjusting by the immigrant status, this association was further supported: adjusted OR (aOR) = 2.68; 95%CI (1.52–4.73), p<0.001.

**Table 2:** Associations between AMA and obstetric background, maternal and neonatal morbidity, and intrauterine fetal death.

	AMA (> = 35 years)		Reference group (24–27 years)								
	n = 1216	%	n = 306	%	p	OR crude	95%	CI	aOR	95%	CI
Obstetric background											
Multiparity (>1) (aOR1)	701	57.65	102	33.33	< 0.001	2.72	2.09	3.54	3.00	2.28	3.94
Repeated abortions >1 (aOR1)	124	10.20	15	4.90	0.004	2.20	1.27	3.82	2.68	1.52	4.73
Maternal morbidity											
Gestational diabetes (aOR2)	43	3.54	4	1.31	0.044	2.77	0.99	7.77	2.70	0.93	7.79
HTN composite (aOR2)	49	4.03	10	3.27	0.537	1.24	0.62	2.48	1.56	0.76	3.18
HBP without preeclampsia (aOR2)	30	2.47	7	2.29	0.855	1.08	0.47	2.48	1.24	0.52	2.93
Preeclampsia (aOR2)	25	2.06	3	0.98	0.211	2.12	0.64	7.07	2.98	0.87	10.21
Instrumental birth (aOR3)	456	37.50	107	34.97	0.412	1.12	0.86	1.45	1.61	1.20	2.17
Cesarean section (aOR3)	318	26.15	68	22.22	0.158	1.24	0.92	1.67	1.58	1.14	2.19
Severe tear (3rd or 4th degree) (aOR3)	2	0.16	2	0.65	0.159	0.27	0.04	1.92	0.21	0.03	1.64
Neonatal morbidity											
Gestational age <37 weeks (aOR1)	110	9.05	21	6.86	0.219	1.35	0.83	2.20	1.28	0.78	2.10
Gestational age <34 weeks (aOR1)	33	2.71	8	2.61	0.918	1.04	0.48	2.28	1.08	0.48	2.40
Apgar <7 at 5 min* (aOR4)	19	1.59	3	0.99	0.441	1.61	0.47	5.48	1.66	0.47	5.81
Apgar <4 at 5 min*	5	0.41	0		0.260						
Low birthweight* (aOR1)	49	4.03	10	3.27		1.18	0.59	2.36	1.07	0.53	2.18
Fetal macrosomia *(aOR1)	47	3.87	21	6.86	0.057	0.54	0.32	0.92	0.61	0.35	1.07
Small for gestational age (aOR1)	26	2.40	6	2.23		1.06	0.43	2.61	1.02	0.41	2.57
Large for gestational age (aOR1)	138	12.72	37	13.75	0.894	0.92	0.62	1.35	1.00	0.67	1.50
Intrauterine fetal death	7	0.58	0		0.183						

*only live fetuses

HTN Hypertension

HBP High Blood Pressure

aOR1: adjusted OR for immigration.

aOR2: adjusted OR for immigration and maternal parity.

aOR3: adjusted OR for immigration, parity, previous cesarean section, and onset of labor (spontaneous or induced).

aOR4: adjusted OR for immigration, parity, previous cesarean section, onset of labor (spontaneous or induced), and instrumental birth (yes/no).

Regarding maternal morbidity, AMA was associated with a greater prevalence of gestational diabetes, OR adjusted by parity and immigration 2.70; 95%CI (0.93–7.79). This association reached statistical significance based on the Chi-Squared test (p = 0.044). The risk of preeclampsia was three times higher among women of AMA, although this association did not reach statistical significance: aOR = 2.98; 95%CI (0.87–10.21).

Lastly, after adjusting for the predefined confounding variables, a statistically significant increase was observed, both for the risk of instrumental delivery, as well as for cesarean sections: aOR for instrumental deliveries = 1.61; 95%CI (1.20–2.17) p<0.001; aOR for cesarean = 1.58; 95%CI (1.14–2.19), p = 0.005.

When restricting the analysis to primiparous women only (Table 3), the strength of the association between the risk of suffering gestational diabetes and AMA increased, with gestational diabetes being eight times more prevalent among primiparous women of AMA: aOR = 8.55; 95CI% (1.12–65.43), p = 0.015.

**Table 3:** Associations between AMA and obstetric history, maternal and neonatal morbidity and intrauterine fetal death; after restricting to primiparous women.

	AMA (> = 35 years)		Reference group (24–27 years)								
	n = 515	%	n = 204	%	p	OR crude	95%	CI	aOR	95%	CI
Obstetric history											
Repeated abortions >1 (aOR1)	40	7.77	8	3.92	0.063	2.06	0.95	4.49	2.87	1.26	6.53
Maternal morbidity											
Gestational diabetes (aOR1)	20	3.88	1	0.49	0.015	8.20	1.09	61.52	8.55	1.12	65.43
HTN composite (aOR1)	33	6.32	8	3.92	0.208	1.65	0.75	3.64	1.64	0.73	3.69
HBP without preeclampsia (aOR1)	17	3.30	5	2.45	0.551	1.36	0.50	3.73	1.32	0.47	3.71
Preeclampsia (aOR1)	21	4.08	3	1.47	0.079	2.85	0.84	9.66	2.81	0.81	9.72
Instrumental birth (aOR2)	281	54.56	88	43.14	0.006	1.58	1.14	2.20	1.63	1.17	2.28
Cesarean section (aOR2)	176	34.17	55	26.96	0.062	1.41	0.98	2.01	1.47	1.02	2.13
Severe tear (3rd or 4th degree) (aOR2)	1	0.19	1	0.49	0.554	0.44	0.03	7.11	0.38	0.02	6.14
Neonatal morbidity											
Gestational age <37 weeks (aOR1)	64	12.43	18	8.82	0.168	1.47	0.85	2.55	1.40	0.80	2.46
Gestational age <34 weeks (aOR1)	16	3.11	7	3.43	0.827	0.90	0.37	2.23	1.04	0.41	2.62
Apgar <7 at 5 min* (aOR3)	14	2.72	2	1.00	0.161	2.78	0.63	12.35	2.12	0.46	9.68
Apgar <4 at 5 min*	3	0.58	0		0.275						
low birthweight* (aOR1)	27	5.26	7	3.43		1.48	0.63	3.46	1.28	0.54	3.02
fetal macrosomia*(aOR1)	12	2.34	11	5.39	0.066	0.42	0.18	0.97	0.46	0.19	1.10
small for gestational age (aOR1)	13	2.90	3	1.72		1.67	0.47	5.95	1.55	0.43	5.66
large for gestational age (aOR1)	42	9.35	19	10.92	0.611	0.85	0.48	1.51	0.90	0.49	1.62
Intrauterine fetal death	2	0.39	0		0.373						

* only live fetuses

aOR1: adjusted OR for immigration.

aOR2: adjusted OR for immigration, previous cesarean and type of onset of labor (spontaneous or induced).

aOR3: adjusted OR for immigration, previous cesarean section, onset of labor (spontaneous or induced), and instrumental birth (yes/no).

The association between repeated abortions, in both crude and adjusted models, was restricted to primiparous women: aOR = 2.87; 95CI% (1.26–6.53). This was also the case for the association with preeclampsia, although without statistical significance: aOR = 2.81; 95 CI% (0.81–9.72).

Likewise, the adjusted associations, both for instrumental deliveries, as well as for cesarean sections, were maintained: aOR for instrumental deliveries = 1.63; 95%CI (1.17–2.28); aOR for cesarean = 1.47; 95%CI (1.02–2.13).

When categorizing AMA based on ordinal ranking into 35–38 years, 39–42 years, and >42, a dose-response pattern was observed in the history of repeated abortions, and the risk of instrumental delivery and cesarean section (the older the age, the greater the OR) (Tables 4 and 5).

**Table 4:** Associations between AMA categorized in an ordinal array and repeated abortions, gestational diabetes and preeclampsia.

	Repeated abortions					Gestational Diabetes					Preeclampsia				
Age	No (n)	Yes (n)	aOR1	95%	CI	No (n)	Yes (n)	aOR2	95%	CI	No (n)	Yes (n)	aOR2	95%	CI
Reference group (24–27 years)	291	15	1			302	4	1			303	3	1		
AMA (ordinal)															
35–38 years	818	65	1.87	1.03	3.39	856	27	2.27	0.77	6.68	865	18	2.89	0.83	10.13
39–42 years	238	49	4.76	2.57	8.84	271	15	4.01	1.28	12.45	281	5	2.49	0.58	10.70
>42	37	10	6.45	2.66	15.66	46	1	1.56	0.17	14.43	45	2	6.12	0.97	38.64
*p trend*			*<0*.*001*					*0*.*036*					*0*.*093*		

aOR1: adjusted OR for immigration

aOR2: adjusted OR for immigration and parity.

**Table 5:** Associations between AMA categorized in an ordinal manner and the risk of instrumental birth, cesarean section and premature birth (<37 weeks).

	Instrumental birth					Cesarean section					Gestational age <37 weeks				
Age	No (n)	Yes (n)	aOR1	95%	CI	No (n)	Yes (n)	aOR1	95%	CI	No (n)	Yes (n)	aOR2	95%	CI
Reference group (24–27 years)	199	107	1			238	68	1			285	21	1		
AMA (ordinal)															
35–38 years	575	308	1.43	1.05	1.94	679	204	1.33	0.94	1.86	796	84	1.36	0.82	2.25
39–42 years	160	126	2.09	1.44	3.04	192	94	2.11	1.42	3.16	262	24	1.19	0.64	2.19
>42	25	22	2.81	1.38	5.71	27	20	4.17	2.05	8.49	45	2	0.57	0.13	2.52
*p trend*			*<0*.*001*					*<0*.*001*					*0*.*921*		

aOR1: OR adjusted by immigration, parity, previous cesarean section and type of onset of labor (spontaneous or induced).

aOR2: OR adjusted by immigration.

Concerning the study of the neonatal and fetal results (prematurity, low Apgar, non-normative birth weight and intrauterine fetal death), no statistically significant positive results were obtained, neither with a strength of association of >1.50, nor for the general sample (Table 2), or when restricted to primiparous women (Table 3).

Lastly, women with AMA had a lower risk of live fetal macrosomia: OR = 0.54; 95%CI (0.32–0.92), and statistical significance was lost after adjusting by immigrant status: aOR = 0.61; 95%CI (0.35–1.07). When restricting to primiparous pregnant women, this protective association was maintained, although without achieving statistical significance.

## Discussion

Our hypothesis was that AMA is associated with maternal and neonatal morbidity. This hypothesis was confirmed as this study revealed that repeated abortions and gestational diabetes were more common among women of AMA. In addition, the possibility of an instrumental delivery or a cesarean was also increased. Lastly, we observed non-statistically significant associations between AMA and preeclampsia, preterm birth (<37 weeks) and low birthweight.

Our results show that a medical history of repeated abortions is up to 2.68 times more common among women with AMA. Furthermore, when performing an ordinal categorization of AMA, a dose-response pattern was observed, with an increased significant linear tendency of repeated abortions as the maternal age increased. This may be because one of the known causes of repeated abortions is chromosomic alterations [27], with evidence of an increased risk of chromosomic alterations with increasing age. Similarly, the results of the cross-sectional study published by Koo YJ et al., in 2012 [17] displayed an OR of 2.7; 95%CI (1.6–4.4), p<0.001 for chromosome alterations and AMA, which increased to 12.3; 95%CI (6.5–23.2, p<0.001 in women with an AMA greater or equal to 40 years. Regarding the association between AMA and a history of abortions, the cohort study by Khalil A et al., in 2013 [28], presents results that support those of our study with an OR of 1.36; 95%CI (1.15–1.62), p<0.001.

Our findings also support a greater prevalence of gestational diabetes in women of AMA. This is especially the case in primiparous women, where the prevalence of maternal morbidity is up to 8.55 times greater. The revised literature which relates gestational diabetes with AMA [5,11,12] supports our findings. In a cross-sectional study focused on primiparous women published in 2015 [2], an OR of 3.58; 95%CI (2.09–5.79), p = 0.0001 was reported. Furthermore, in the study by Heras B et al., published in 2011 [11], comparing women both older and younger than 35 years, the authors reported an OR of 3.66; 95%CI (1.50–8.91). These results are supported by a recently published cross-sectional study [18], both in the group of 35–39 years: OR 1.15; 95%CI (1.01–1.27) as well as in the group of >40 years: OR 2.41; 95%CI (2.13–3.76). The causes of gestational diabetes continue to be a subject under research. One of the theories is that, in a normal pregnancy, the stimulus of placental lactogen and prolactin produce a pancreatic hyperplasia of the B cells. At the same time, diabetogenic hormones, such as the growth hormone, the hormone that liberates corticotropin, placental lactogen and progesterone, produce an increased insulin resistance. When this insulin resistance cannot be overcome, despite the hyperplasia of B cells, gestational diabetes occurs [29]. The potential influence of maternal age on the development of gestational diabetes continues to be a subject of research and requires further clarification and study.

We found a three-fold greater risk of preeclampsia among women with AMA. This factor was reflected both in the total sample and when restricted to primiparous women, although the latter association did not reach statistical significance. One of the possible limitations of this study is the lack of statistical power. Therefore, certain associations may not reach statistical significance, especially due to the low prevalence of these morbidities (only 1% in women aged 24–27 years, according to our results). A meta-analysis of 22 articles conducted on a total of 5,244,543 women, supports this association, reporting an OR of 1.2; 95%CI (1.1–1.3) [30].

In terms of instrumental deliveries, our results suggest an association with AMA, as the risk of instrumental deliveries was up to 1.61 times greater among AMA pregnant women overall, and with a clear dose-response pattern (the older the woman, the greater the risk), thus, pregnant women aged over 42 years old had a 2.81 times greater risk of instrumental delivery when compared to pregnant women aged between 24–27 years. When restricting to primiparous women, the same strength of association was obtained: adjusted OR = 1.63; IC95% (1.17–2.28). In 2015, Schimmel MS et al., [2], also reported a similar result concerning primiparous women: OR = 1.59; 95%CI (1.19–2.14).

Lastly, concerning maternal morbidity, our results suggest a greater risk of cesarean births among women with an AMA with a clear dose-response pattern, with cesarean sections being 4.17 times more common in pregnant women over the age of 42 years. The results of a cross-sectional study [2] were similar to those obtained in our study, when comparing the group with AMA to the same group of reference aged 24–27 years, as used in our study, reporting an OR of 2.46; 95%CI (1.65–3.677), p<0.001. When dividing the group with AMA into ordinal subgroups by age, a dose-response pattern was also obtained. Some studies also reveal a relationship between AMA and an increase in the percentage of cesarean sections [10–14]. There is considerable controversy regarding the causes behind suffering an increased risk of a cesarean among women of AMA. In a systematic review performed where this association was studied, the most common explanation was an inefficiency of the aged myometrium. This would be in addition to a decrease in the number of oxytocin receptors and could lead to a cesarean section as effective uterine dynamics were not achieved for dilation and delivery [13].

The neonatal results for prematurity reveal mixed results for AMA, with studies that do not support this association or which present statistically significant results [2, 11, 18, 19]. This is concurrent with other studies, such as a paper by Koo YJ et al 2012 [17], obtaining significant results for births <37 weeks of gestation: OR 1.4 95%CI (1.2–1.7) p<0.001 and preterm births <32 weeks’ gestation: OR 1.9 95%CI (1.3–2.7) p<0.001. Thus, in a cross-sectional study published in 2015 [20], a significant association was observed for women >41 years. Our results for the 37-week cut-off reveal a prevalence of preterm births up to 1.5 times greater in women of AMA, although this association did not achieve statistical significance, possibly due to the lack of statistical power mentioned previously. Therefore, future primary studies or meta-analyses should also study this association.

Relating to the low Apgar score at five minutes, no cases registered a score below four in the group of pregnant women aged between 24–27 years, therefore an odds ratio association was not possible. The differences in percentages did not achieve statistical significance because of the low prevalence. This result is in agreement with previous findings [2, 17, 18].

Regarding the low birth weight, we found a positive association (AMA as a risk factor). However, the magnitude of this association was low and did not yield statistical significance. This coincides with the results reported in an article by Heras B et al., published in 2011 [11]. When restricted to primiparous women, our positive association remained albeit without statistical significance. Schimmel [2] also reported a positive association in primiparous women albeit without statistical significance: OR 1.65, 95%CI (0.98–2.77), p = 0.058.

According to our results, there was a lesser prevalence of fetal macrosomia with AMA, with the association being, therefore, negative as a protective factor. These results must be interpreted with caution as, curiously, among the 24–27 years age group, selected as the reference, the prevalence of fetal macrosomia was 7.8%, greater than in women under the age of 24 where the prevalence was 3.4%, and also, when compared to the group that was immediately older (28–34 years), where the prevalence was 6.4%, although this could be purely coincidental. Upon comparing these findings with the international literature available, a former cross-sectional study also found an opposite association whereby AMA was associated with an increased risk of newborns large for gestational age: OR 1.64; 95%CI (1.51–1.79), p<0.001 [2].

In terms of implications for clinical practice, our results support the available evidence between AMA and maternal and neonatal morbidity. This should be reflected in a more individualized and comprehensive gestational control of these women. Initially, the preconception consultation should include a specific preconception risk assessment specifically focusing on the risks related to AMA. During gestation, given the increased risk of gestational diabetes, an early screening in the first trimester may be recommended, especially in primiparous women, together with preventive and health education and promotion activities insisting on the importance of diet and physical exercise. In the case of pre-eclampsia, the sum of risk factors such as AMA, personal history, or obesity should be considered. When several of these factors are present in the same woman, this may require an early and more exhaustive screening, thus emphasizing once again the importance of diet and physical exercise and including weight control. Lastly, in relation to fetal monitoring, an increase in the number of ultrasound scans to assess fetal growth may be advisable.

In retrospective studies such as the present study, where data is based on secondary information (medical records), one of the main limitations may be the poor quality of the information, which could lead to a possible information bias. To minimize such bias, prior to the onset of the study, we selected the variables which tend to be stated more homogenously, systematically and objectively in the medical records. However, it is important to note a further limitation, as it was not possible to identify certain variables homogeneously and systematically, such as the weight or BMI of the pregnant women; their educational level; or the pregnancies as a result of treatment with reproductive technologies. These variables could potentially be associated with both AMA and maternal and neonatal morbidity acting as confounding variables. The fact that we were able to study all the births occurring during the study period would minimize the possibility of a selection bias. The study population represents 90% of all the births attended in the autonomous community of Cantabria (Northern Spain) within the public health system (according to the official data of births for the year 2014) and 73% of the births if we were to include the births attended in the private health sector [31]. This also supports the external validity of our results.

## Conclusion

Our results show an association between AMA and a history of repeated abortions. Regarding maternal morbidity, AMA was associated with a greater risk of suffering from gestational diabetes, especially among primiparous pregnant women, as well as being associated with instrumental deliveries and cesareans. Although our results were not statistically significant, an association between AMA and a greater risk of preeclampsia is suggested.

In terms of a greater risk of neonatal morbidity, our results suggest an association between a low birth weight and preterm births < 37 weeks, although these associations did not reach statistical significance.
